# Supplementary material for: Identifying Predictors for the Acquisition of Tolerance to Cow’s Milk Protein in Infants with Food Protein-Induced Allergic Proctocolitis (FPIAP): Multifactorial Analysis of Two Italian Cohorts
Source: Nutrients. 2025 Dec 27;18(1):95. doi: 10.3390/nu18010095 (PMC12787599; doi:10.3390/nu18010095)
Supplement: Supplementary file 1 [file nutrients-18-00095-s001.zip › nutrients-4022127-supplementary.pdf]

**Supplementary Table S1:** Factors that may potentially function as predictors of late tolerance acquisition to cow's milk in Varese cohort

| | **Odds ratio (OR)** | **Odds ratio (OR) 95%CI lower bound** | **Odds ratio (OR) 95%CI upper bound** | **p-value** |
|---|---|---|---|---|
| **family history of atopy** | 1.016×10+25 | 0.000 | ∞ | 0.997 |
| **atopic dermatitis** | 2.539×10+25 | 0.000 | ∞ | 0.997 |
| preterm delivery | 6.174×10+27 | 0.000 | ∞ | 0.000 |
| micronutrient fortification of milk in the first weeks of life | 4.645×10+8 | 0.000 | ∞ | 0.000 |
| growth faltering | 2.340×10-25 | 0.000 | ∞ | 0.997 |
| **rectal bleeding duration >18 days before starting elimination diet** | 7.592×10+16 | 0.000 | ∞ | 0.998 |
| antibiotic treatment within the first seven days of age | 0.038 | 0.000 | ∞ | 0.000 |
| recurrent infections | 2.180×10-8 | 0.000 | ∞ | 0.998 |
| **IgE sensitization** | 4.317×10-11 | 0.000 | ∞ | 0.000 |

**Supplementary Table S2**: Factors that may potentially function as predictors of late tolerance acquisition to cow's milk in Milan cohort

| | **Odds ratio (OR)** | **Odds ratio (OR) 95%CI lower bound** | **Odds ratio (OR) 95%CI upper bound** | **p-value** |
|---|---|---|---|---|
| **family history of atopy** | 0.513 | 0.055 | 4.815 | 0.559 |
| **atopic dermatitis** | 7.369×10-8 | 0.000 | ∞ | 0.997 |
| preterm delivery | 1.378 | 0.020 | 94.632 | 0.882 |
| micronutrient fortification of milk in the first weeks of life | 0.981 | 0.021 | 45.661 | 0.992 |
| growth faltering | 3.984 | 0.067 | 236.884 | 0.507 |
| **rectal bleeding duration >18 days before starting elimination diet** | 2.279×10-8 | 0.000 | ∞ | 0.996 |
| antibiotic treatment within the first seven days of age | 0.309 | 0.008 | 11.402 | 0.523 |
| recurrent infections | 2.947 | 0.140 | 61.908 | 0.487 |
| **IgE sensitization** | 1.549 | 0.064 | 37.646 | 0.788 |
